# A methodology for small area prevalence estimation based on survey data

**DOI:** 10.1186/s12939-020-01220-5

**Published:** 2020-07-31

**Authors:** Regina Tomie Ivata Bernal, Quéren Hapuque de Carvalho, Jill P. Pell, Alastair H. Leyland, Ruth Dundas, Mauricio Lima Barreto, Deborah Carvalho Malta

**Affiliations:** 1 Escola de Enfermagem, Universidade Federal de Minas Gerais (UFMG). Departamento de Enfermagem Materno-Infantil e Saúde Pública, Belo Horizonte, Minas Gerais, Brazil; 2 Institute of Health and Wellbeing, University of Glasgow, Lilybank Gardens, Glasgow, G12 8RZ UK; 3 Medical Research Council/Chief Scientific Office (MRC/SCO) Social and Public Health Sciences Unit, Glasgow, Scotland; 4 Universidade Federal da Bahia. Instituto de Saúde Coletiva, Salvador, BA Brazil

**Keywords:** Small area estimation, Telephone survey

## Abstract

**Background:**

Brazil conducts many health surveys to provide estimates by national level, macro-regions, states, metropolitan regions and capitals. However, estimates for smaller areas are lacking due to their high cost. The Health Vulnerability Index (in Portuguese, Índice de Vulnerabilidade em Saúde, IVS) is a measure that combines socioeconomic and environmental variables in the same indicator and allows for the analysis of the characteristics of population groups residing in census tracts, grouping them into four health risk areas (low, medium, high and very high risk) in addition to showing inequalities in the epidemiological profile of different social groups. This index was developed by the Municipal Health Secretariat of Belo Horizonte to guide health planning.

**Objective:**

The aim of the study is to produce a methodology for obtaining reliable estimates for tobacco smoking in small areas for which the IVS was not designed.

**Methods:**

The Vigitel dataset from 2006 to 2013 was used to obtain estimates of the prevalence of smokers based on the IVS employing small area estimation methods that use data from a larger domain to obtain estimates in smaller areas. For indirect estimates, the covariates included were sanitation, housing, education, income, and social and health factors. Post-stratification weights were used according to the IVS based on the population of the 2010 census.

**Results:**

From 2006 to 2009, 16.2% (95% CI: 13.6–14.8%) of the adult population in Belo Horizonte were smokers, and 14.8% (95% CI: 14.0–15.6%) were smokers between 2010 and 2013. The very high-risk population maintained a high prevalence over the same period of 21.1% (95% CI: 17.1–25.0%) between 2006 and 2009 and 20.8% (95% CI: 17.0–24.6%) between 2010 and 2013, while in the low-risk group, the prevalence in the same period fell from 14.9% (95% CI: 13.7–16.2%) to 11.8% (95% CI, 10.6–13.1%).

**Conclusions:**

The present study identified differences in the profile of smokers by the IVS in the city of Belo Horizonte. While the smoking prevalence declined in richer areas, it remained high in poor areas. This methodology can be used to produce reliable estimates for subgroups with greater vulnerability in small areas and thus subsidize the formulation, monitoring and evaluation of public health policies and programmes aimed at smoking.

## Background

The repercussions caused by social inequalities in the field of public health have been studied for a long time. Such studies aim to understand the interrelationship between social, economic and epidemiological indicators, considering that people in disadvantaged circumstances and those living in areas of greater vulnerability almost invariably have worse health indicators and outcomes [1]. It is important to understand and monitor health inequalities if the Sustainable Development Goals (SDG) [2] are to be achieved.

Non-communicable diseases (NCDs) are more prevalent in economically disadvantaged populations and result in a great impact on health systems and on individual and collective quality of life [3]. The four main risk factors for NCDs are sedentary behaviour, high caloric intake, excessive consumption of alcohol, and tobacco use. The harmful effects of smoking are widely documented in global and national studies [4, 5]. According to the World Health Organization (WHO), tobacco constitutes the main risk factor for preventable causes of death and the second largest attributable factor for mortality in the world [5, 6].

For the SDGs to be met, no population group can be left behind. In this regard, in recent decades, place of residence has been strongly associated with an individual’s socioeconomic circumstances, suggesting that various aspects of population characteristics, economic status, and living conditions in the neighbourhood may be important for the perpetuation of inequities [7].

In addition to considering different social aspects, epidemiological research uses spatial analysis to identify the influence of spaces on different levels of exposure and inequalities, expanding the understanding of the occurrence of health-related events in populations and processes of morbidity and mortality [8, 9].

Spatial analyses enable the identification of the explanatory chain of the health-disease process according to the territorial reality and are essential for the orientation of intersectoral policies and actions in various places [9]. As their main objective, these policies should seek to overcome the inequalities generated by the unequal distribution of resources and should go beyond individual contexts to be implemented in the planning of urban spaces and contexts [1, 9].

Area-level measures that capture social vulnerabilities, material deprivation or human development have been developed for many different countries [10–12]. These measures are available for the whole population regardless of age, gender and other characteristics and are useful when individual measures of socioeconomic position are not readily available. These area-level measures are used extensively in research to understand and describe inequalities in health and mortality and are increasingly used to inform policy and target resources [12]. The Municipal Health Secretariat of Belo Horizonte, Brazil, created a composite indicator, the Health Vulnerability Index (in Portuguese, Índice de Vulnerabilidade em Saúde, IVS), at the small area (census sector) level. This has been used as an important strategy in the identification of areas of vulnerability [13].

Population surveys are an effective and reliable tool to collect data on NCD risk factors, but their use to understand small area variation in such risk factors is limited. This is due to the small number of survey respondents in each small area. Another weakness of using a survey sample is that the estimates of prevalence may be biased if the survey is not representative of the population. Post-stratification weights can improve such population prevalence estimates. The use of composite indicators such as the IVS can support the production of estimates related to risk factors for NCDs and thus support policies for the promotion of equity [13].

The aim of this study is to produce a methodology for obtaining reliable estimates of tobacco use in small areas for which the sample design was not planned.

## Methods

This is a cross-sectional study using data from the Brazilian Surveillance of Risk and Protective Factors for Chronic Diseases through Telephone Interviews (Vigitel) for the municipality of Belo Horizonte over the period from 2006 to 2013 [14–21].

Vigitel is conducted annually in 26 Brazilian state capitals and in the Federal District, and the study population comprises adults aged 18 years and over. In the sampling process, Vigitel uses the registers of residential telephones to draw household samples. According to the 2010 census, 61.0% of the private households located in the 26 capitals and the Federal District had at least one landline. Due to low landline coverage, Vigitel uses post-stratification weights according to age, gender and schooling to compensate for this low coverage and thus reduce potential bias.

### Dataset

Vigitel data from 2006 to 2013 collected for Belo Horizonte were used to estimate the prevalence of smoking, and the IVS instrument was used to estimate the prevalence for small areas based on the Vigitel sampling design. Vigitel uses post-stratification weights to adjust the population of the 2010 census by the IVS. The post-stratification weights are obtained by the rake method [22]. This method works with one variable at a time, corresponding to the total distributions of the variable in the sample and in the population through iterative procedures. This process is then repeated for each of the variables used in the construction of weights so that the sample distribution becomes identical to the population distribution for these variables.

This study used the IVS to estimate smoking prevalence for small areas. This index is a measure that combines socioeconomic and environmental variables in the same indicator and allows for the analysis of the characteristics of population groups residing in census tracts (which are the smallest territorial division adopted by the Brazilian Institute of Geography and Statistics, IBGE) [23]. The IVS was developed by the Municipal Health Secretariat of Belo Horizonte in 1998 with the objective of guiding the planning of health actions and was updated in 2012 using data from the 2010 census; this was the version used in the present study. The IVS enables the highlighting of inequalities between areas, and in 2012, it included the following variables from the 2010 census: sanitation components, defined as the proportion of households with inadequate sewage conditions (no bathroom or type of drainage system for toilets, such as septic tank, rudimentary fossa, or another sewer), proportion of households with inadequate waste disposal (burned, buried and thrown in river, lake or sea or any other way), proportion of households with inadequate water supply (without general network, spring with internal piping, well or any other way), proportion of illiterate people, average household size and proportion of black, brown or indigenous people. The indicator was normalized $\left({Indicator}_{new=}{I}_n=\frac{Indicator-{Indicator}_{min}}{Indicator_{max}-{Indicator}_{min}}\right)$ into the range [0,1] and collapsed into four clusters: low (I_n_ ≤ 0.1957), medium (0.1957 < I_n_ ≤ 0.2865), high (0.2865 < I_n_ ≤ 0.3782) and very high (I_n_ > 0.3782). According to the 2010 census, Belo Horizonte had 2.4 million inhabitants living in 3830 census tracts, which were grouped based on the IVS into four clusters of health risk: low (1330 census tracts), medium (1460 census tracts), high (737 census tracts) and very high risk (303 census tracts) [13].

### Geoprocessing

The first step was to include the census tracts in the Vigitel databases using the linkage method with the National Register of Addresses for Statistical Purposes of the 2010 census [23] and the registrations of addresses of landline phones. The second step was to include IVS information by census tract.

The Vigitel database was divided into two periods, 2006 to 2009 and 2010 to 2013, and the proportion of adult smokers based on the IVS was estimated in each period. One or more Vigitel interviews occurred in 2803 (73%) census tracts in the first period and 2790 (73%) in the second period out of a total of 3830 census tracts.

### Small area estimation

The historical series of Vigitel showed a downward trend in the frequency of adult smokers in the period from 2006 to 2013. Estimates were calculated according to the IVS using direct and indirect methods employing post-stratification weights. The methodology of Rao and Molina (2015) [24] was employed for small area estimation, and two alternative methods were used.

#### Direct estimation

Direct estimation for small areas entails the use of sample design variables to obtain the estimates for smaller areas. This study used Vigitel data in the 2006 to 2013 period and defined sample weights as $\left( weight=\frac{number\ of\ adults\ in\ the\ household}{number\ of\ landline\ phones\ in\ the\ houshehold}\right)$.

For the joint analysis of Vigitel data, the calculation of post-stratification weights was necessary to adjust the Vigitel sample to the population of 2010 according to the IVS using the Rake method [22]. This method uses only the marginal frequencies of each variable of the population and the sample. It works with one variable at a time, matching the total distribution of the variable in the sample to that in the population, weighted using the sample weights, by iterative procedures. This process is then repeated for each of the variables used in the construction of weights so that the sample distribution becomes identical to the population distribution for these variables. Weights were calculated in STATA using the SURVWGT [25] package, where post-stratification weights were constructed for the periods 2006 to 2013, 2006 to 2009 and 2010 to 2013. The reference population used in the calculation of post-stratification weights was extracted from the 2010 census of Belo Horizonte.

#### Indirect estimation

Indirect estimation involves the use of statistical models to obtain estimates of the proportions of adult smokers. A Vigitel dataset with only one interview by census tract was used to build a logistic regression model to estimate the response variable Smoker (Y), yes (1) or no (0) in the set of census tracts without any interviews. The distribution of sectors with one single interview by IVS is similar to the distribution of sectors without any interview. Thus, it was possible to estimate the probability of one individual in each census tract being classified as a smoker or non-smoker for the set of census tracts without any Vigitel interviews. There were 1027 census tracts without any interviews in the 2006 to 2009 period and 1040 census tracts in the period from 2010 to 2013.

To construct the model, census tracts with a single interview (535 census tracts) were selected, with the dichotomous response variable (y_i_) taking the value of 1 for smokers and 0 otherwise. The covariates for each census tract were extracted from the 2010 census, such as the percentage of households by type of water supply, percentage of households by type of sanitary sewage, percentage of households without the presence of male persons, percentage of households with women heads of household aged 16–30 years old, percentage of households with the presence of great-grandchildren between 0 and 14 years of age, percentage of households including sisters or brothers above 50 years and percentage of households with six or seven residents.

For the modelling process, the sample of 535 census tracts was divided into two subsamples: the first with 261 census tracts was used for the construction of the logistic regression model, and the second with 274 census tracts was used to validate the model to ensure that the model obtained in the first sample was robust.

The general logistic regression model [26] is given by:

$$ \log \kern0em \left\{\frac{\pi (x)}{1-\pi (x)}\right\}={\beta}_1\kern0.5em +\kern0.5em {\beta}_2{x}_2\kern0.5em +\kern0.5em \dots +\kern0.5em {\beta}_p{x}_p, $$

where:

*x* = (*β*_1_, *β*_2_…, *β*_*p*_)is the vector of the covariates;

is the probability that the respondent is a smoker given a set of covariates;

*β* = (*β*_1_, *β*_2_…, *β*_*p*_)is the vector of coefficients.

Census tracts with a predicted probability greater than or equal to 0.13 (the threshold) were classified as smokers and otherwise as non-smokers (probability < 0.13). This corresponds to a 13% prevalence of regular or occasional smoking in the Vigitel interview. This value was obtained from the set of census tracts with a single Vigitel interview.

To evaluate the model adjustment, we used a two-by-two classification matrix. The true positive (TP) denotes a response of smoking being correctly classified by the model; the true negative (TN) denotes a response of non-smoking being correctly classified as non-smoking. False negative (FN) responses were classified as no smoking, and false positive (FP) responses were classified as no smoking. The sensitivity of the model is defined by TV/(TV+ FN), the specificity is defined by TN/(TN + FP), and the accuracy is measured as (TP + TN)/(TP + FN + TN + FP).

In the joint analysis of census tracts with and without interviews, the post-stratification weight adjusted for the population of the 2010 census by the IVS was calculated using the Rake method. These weights were calculated in STATA using the SURVWGT [25] package, and the sample weight information was required to execute the package. In this study, we considered the data of populations N_1_ and N_2_ extracted from the 2010 census of Belo Horizonte to calculate the weight of the group of census tracts with Vigitel interviews $\left( weight=\frac{N_1}{n_1}\right)$ and without interviews $\left( weight=\frac{N_2}{n_2}\right)$, where N_1_ is the population of adults in the census tracts with interviews, N_2_ is the population of census tracts without interviews, n_1_ is the number of census tracts with Vigitel interviews, and n_2_ is the number of census tracts without interviews.

## Results

**Fig. 1:**
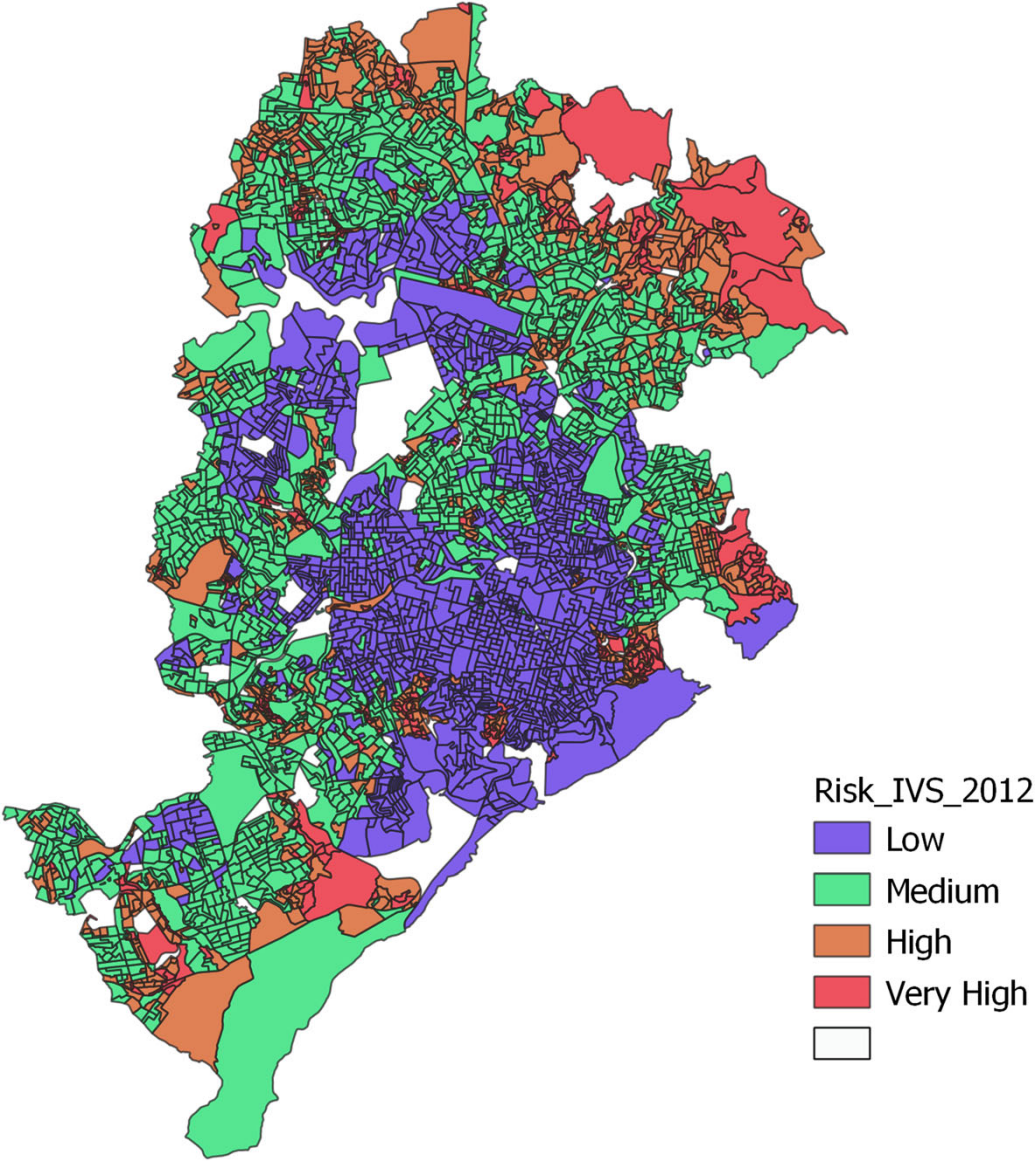
Map of Belo Horizonte City by census tract, ranked by Health Vulnerability Index (IVS)

From the total of 15,833 interviews conducted in the 2006 to 2013 period, the census tracts of 13,663 (86%) could be identified. Respondents were drawn from 3317 (87%) out of a total of 3830 census tracts, indicating the sparsity of responses in most census tracts. In terms of representativeness of the Vigitel census tracts according to the IVS, these census tracts represent 91.2% (1213) and 88.9% (1298) of the low- and medium-risk groups and 79.8% (588) and 71.9% (218) of the high- and very-high-risk groups (out of a total of 1330 low-, 1460 medium-, 737 high- and 303 very-high-risk census tracts) (Fig. 1).

In the 2006 to 2013 period, 513 (13.4%) census tracts did not contain any Vigitel interviews, 535 (14.0%) had only one interview, and 130 (3.4%) census tracts contained 10 or more interviews. In the periods 2006 to 2009 and 2010 to 2013, the number of census tracts without interviews was higher than over the full period, as was the number of census tracts with a single interview (Table 1).

**Table 1:** –Distribution of number of interviews by census tracts of period

Number of interviews	2006 to 2013		2006 to 2009		2010 to 2013	
	Number of census tracts	%	Number of census tracts	%	Number of census tracts	%
0	513	13.4	1027	26.8	1040	27.2
1	535	14.0	957	25.0	948	24.8
2	523	13.7	720	18.8	770	20.1
3	528	13.8	513	13.4	509	13.3
4	473	12.3	314	8.2	292	7.6
5	389	10.2	178	4.6	146	3.8
6	293	7.7	64	1.7	70	1.8
7	214	5.6	33	0.9	34	0.9
8	138	3.6	15	0.4	10	0.3
9	94	2.5	6	0.2	8	0.2
10	55	1.4	3	0.1	–	–
11	41	1.1	–	–	2	0.1
12	16	0.4	–	–	–	–
13	9	0.2	–	–	–	–
14	2	0.1	–	–	–	–
15	5	0.1	–	–	1	–
17	1	0.0	–	–	–	–
23	1	0.0	–	–	–	–
Total	3830	100	3830	100.0	3830	100.0

The Vigitel survey responses varied by the IVS, with more survey responses in the lower and medium IVS risk groups and fewer in the high and very high risk groups. The distribution was similar across the two time periods (Table 2).

**Table 2:** Distribution of the number of census tracts by period and IVS by Vigitel

Period	IVS	Vigitel interviews					
		No		Yes		Total	
		n	%	n	%	n	%
2006 to 2013	Low	117	8.8	1213	91.2	1330	100.0
	Medium	162	11.1	1298	88.9	1460	100.0
	High	149	20.2	588	79.8	737	100.0
	Very High	85	28.1	218	71.9	303	100.0
	Total	513	13.4	3317	86.6	3830	100.0
2006 to 2009	Low	254	19.1	1076	80.9	1330	100.0
	Medium	345	23.6	1115	76.4	1460	100.0
	High	279	37.9	458	62.1	737	100.0
	Very High	149	49.2	154	50.8	303	100.0
	Total	1027	26.8	2803	73.2	3830	100.0
2010 to 2013	Low	304	22.9	1026	77.1	1330	100.0
	Medium	358	24.5	1102	75.5	1460	100.0
	High	245	33.2	492	66.8	737	100.0
	Very High	133	43.9	170	56.1	303	100.0
	Total	1040	27.2	2790	72.8	3830	100.0

In the 2006 to 2013 period, 14.5% (95% CI: 13.8–15.2%) of the adult population were smokers based on Vigitel data from Belo Horizonte. However, this frequency fell over time, from 15.71% (95% CI: 13.75–17.68%) to 12.77% (95% CI: 10.77–14.77%) (Table 3).

**Table 3:** Estimated prevalence history series of adult smokers, Vigitel 2006 to 2013

Year	%^a^	95% CI
2006	15.71	(13.75;17.68)
2007	14.98	(13.00.16.96)
2008	16.43	(14.38;18.49)
2009	14.34	(12.49;16.19)
2010	15.05	(13.08;17.01)
2011	14.58	(12.69;16.47)
2012	12.47	(10.63;14.31)
2013	12.77	(10.77;14.77)
Mean	14.52	(13.83;15.21)

^a^Sex, age and schooling adjusted for the population of the current year of the research

### Estimation by IVS: direct method

In the 2006 to 2013 period, it is noted that this proportion was higher in the population belonging to the very-high-risk group at 15.32% (95% CI: 12.15–18.48%), followed by 13.98% (95% CI: 12.36–15.60%) in the high-risk group., this proportion was 13.17% (95% CI: 12.17–14.18%) in the low-risk group and 13.69% (95% CI: 12.71–14.68%) among those at average risk. However, it is noteworthy that there is no statistically significant difference between the low-, medium-, high- and very-high-risk groups (Table 6). The increasing gradient in the proportion of adult smokers estimated by the IVS observed in the full period is not present in the period from 2006 to 2009. In the 2010 to 2013 period, the proportion of adult smokers estimated by the IVS maintains the pattern found across the full period. In the comparison between 2006 and 2009 and 2010 to 2013, a decrease in the proportion of adult smokers in the low-risk group was noted, from 15.04% (95% CI: 13.54–16.42%) to 11.02% (95% CI: 9.69–12.36%). The medium-risk and high-risk groups did not show significant variation between the periods, while the prevalence among the very-high-risk group increased from 13.71% (95% CI: 9.39–18.05%) to 16.64% (95% CI: 12.10–21.18%) (Table 6).

### Estimation by IVS: indirect method

According to the adjusted logistic regression model, the probability of an adult living in the census tract being classified as a smoker is very low since the model constant is − 3.878; that is, the chance of an adult being classified as a smoker is equal to 0.021 (exponential of β). Eight of the variables associated with smoking increased the probability of the census tract being classified as a smoker, and only the variable presence of women heads of household aged 16 to 30 years decreased the probability of the census tract being classified as a smoker (Table 4).

**Table 4:** Logistic regression analysis of smoking

Independent Variable	Coefficient	SE	***p***-value	Exp(B)
% household type of house - condominium house	0.016	0.005	0.001	1.016
% household well water supply	0.029	0.025	0.240	1.030
% household water supply for tankers	1.896	1.490	0.203	6.656
% households sanitary primary sewage	0.033	0.023	0.154	1.033
% households 6 to 7 residents	0.057	0.035	0.099	1.059
% households no male	0.049	0.021	0.029	1.051
% female heads of household aged 16 to 30	−0.038	0.017	0.029	0.963
% great-grandson or great-granddaughter of household aged 0 to 14	0.017	0.007	0.018	1.017
% son- or daughter-in-law in household aged 50+	0.013	0.008	0.098	1.013
Constant	−3.878	0.744	0.000	0.021

In the evaluation of the model adjustment, the cut-off point for classification of the census tracts as smokers or non-smokers was 13%. This value was obtained from the sample of 535 census tracts. In sample 1, the mean accuracy of the model was 66.7%, with sensitivity equal to 53.7% and specificity of 69.1%. In sample 2, the average hit was 63.1%, with a sensitivity of 66.9% and specificity equal to 65.6%. It is to be expected that the mean accuracy of sample 2 is lower than that of sample 1. However, the sensitivity in sample 2 is better than in sample 1, and consequently, the specificity in sample 1 is lower than in sample 2. These results show that the adjusted logistic regression model is adequate (Table 5).

**Table 5:** Accuracy of the logistic regression model

Sample	Dependent variable	Smoker (model estimate)				Total
		0 (No)		1 (Yes)		
	Smoker	n	%	n	%	
**1**	0 (No)	152	69.1	68	30.1	220
	1 (Yes)	19	46.3	22	53.7	41
	Total	171	65.5	90	34.5	261
**2**	0 (No)	162	66.9	80	33.1	242
	1 (Yes)	11	34.4	21	65.6	32
	Total	173	63.1	101	36.9	274
**Total**	0 (No)	314	68.0	148	32.0	462
	1 (Yes)	30	41.1	43	58.9	73
	Total	344	64.3	191	35.7	535

There was some difference between the two time periods in the prevalence of smoking by the IVS using the direct method. In the 2010 to 2013 period, the low-risk group presented a lower prevalence (− 2.55, 95% CI:-4,48%;-0.60%) of smoking adults when compared to the medium group and − 5.61% (95% CI:-10.34%;-0.08%) when compared to the very-high-risk group. On the other hand, the estimates obtained by the indirect method in the 2006 to 2013 period show significant differences between the low- and high-risk groups. In the periods from 2006 to 2009 and 2010 to 2013, the high- and very-high-risk groups presented the highest prevalence of adult smokers when compared to medium- and low-risk groups (Table 6). In the comparison between the estimates of adult smokers in the periods 2006 to 2009 and 2010 to 2013, it is noted that in both methods of estimation in small areas, there is a decreasing trend observed in Table 3. However, the magnitudes differ. The direct method estimates between 14.49% (95% CI: 13.56–15.42%) and 12.73% (95% CI: 11.85–13.60%), while the indirect method estimates between 16.18% (95% CI: 15.35–17.01%) and 14.81% (95% CI: 14.00–15.61%). In the comparison between the periods by the IVS, the direct method estimates show that the prevalence of adult smokers was lower in the low-risk group (− 4.02, 95% CI: − 6.01%; − 2.03%), and in the other groups, the differences were not significant. The estimates obtained by the indirect method also show lower levels of smoking in the low-risk group (− 3.10, 95% CI: − 4.86%;-1.34%). Despite the differences between the estimates obtained from the direct and indirect methods in the period 2010 to 2013, both indicate that the prevalence of smoking in adults is higher in the very-high-risk group, but with different magnitudes.

**Table 6:** Estimated prevalence of adult smokers by period and by the IVS according to small area method, Vigitel 2006–2013

Period	IVS	Small Area Method			
		Direct		Indirect	
		% smoker	CI (95%)	% smoker	CI (95%)
2006 to 2013	Total	13.65	(13.02;14.29)	14.14	(13.65;14.83)
	Low	13.17	(12.17;14.18)	13.43	(12.51;14.35)
	Medium	13.69	(12.71;14.68)	14.00	(13.10;14.91)
	High	13.98	(12.36;15.60)	15.79	(14.23;17.35)
	Very High	15.32	(12.15;18.48)	16.23	(13.42;19.06)
2006 to 2009	Total	14.49	(13.56;15.42)	16.18	(15.35;17.01)
	Low	15.04	(13.54;16.52)	14.97	(13.69;16.24)
	Medium	13.82	(12.42;15.21)	15.11	(13.84;16.38)
	High	15.14	(12.66;17.62)	19.42	(17.17;21.66)
	Very High	13.71	(9.39;18.05)	21.09	(17.12;25.05)
2010 to 2013	Total	12.73	(11.85;13.60)	14.81	(14.00;15.61)
	Low	11.02	(9.69;12.36)	11.87	(10.65;13.08)
	Medium	13.57	(12.18;14.97)	15.42	(14.13;16.71)
	High	13.00	(10.87;15.13)	17.46	(15.38;19.53)
	Very High	16.64	(12.10;21.18)	20.78	(16.97;24.60)

(*) adjusted by 2010 census population adults by IVS.

## Discussion

This study proposes a methodology for obtaining reliable estimates for tobacco prevalence in small areas that were not planned in survey design. The indirect method indicates that the prevalence of smoking is higher in the high-risk group than in the low-risk group.

Studies in Brazil have considered spatial analysis and its relations with health, such as the Adult Health Survey, in the metropolitan Region of Belo Horizonte (Minas Gerais), which sought to evaluate the perception of a neighbourhood’s social environment and self-assessed morbidity, the health study in Belo Horizonte that investigated the psychometric qualities of contextual characteristics measured by the perception of the social and living conditions of residents participating in the survey, and the association with physical activity at different socioeconomic levels [27].

Bernal and Silva (2009) [28] showed that landline coverage is not evenly distributed in the population, and users are concentrated in the most favourable social classes. In addition, landline ownership is associated with schooling and skin colour. These findings corroborate the results found in this study by the IVS since most of the census tracts identified in Vigitel are concentrated in the low- and medium-risk health groups.

The estimation from the spatial analysis allowed us to identify inequalities in the health districts of Venda Nova and Barreiro in Belo Horizonte, such as a higher proportion of alcohol abuse and whole milk consumption, low regular consumption of fruits and vegetables and less activity practice in free time. These results can support planning aimed at actions for greater equity in health [29]. Spatial correlation analysis identified neighbouring areas with similar health behaviours and similar underlying social, economic and cultural characteristics. Social, economic, cultural and health outcomes sometimes behave in a heterogeneous way, creating differences that require the actions of managers to direct health policies to reach the different social groups living in an area [1].

The use of the IVS allows for the aggregation of the spatial analysis of health outcomes, notably, estimating the prevalence of smoking. The use of composite indicators has resulted in innovation in the identification of health inequalities [13]. The Human Development Index (HDI) composite indicator developed by the United Nations assesses the quality of life and economic development of the population and has enabled the flow of international resources to be directed to the nations with greatest need [30]. The Sociodemographic Index (SDI) used by the Global Burden of Disease study enables the identification of inequalities between countries and at a subnational level, showing the importance of aggregating social covariates to explain health outcomes according to sociodemographic differences [31].

Composite indicators that are easily accessible and interpretable, such as the IVS, are important tools when redesigning a network of assistance and promoting population development. In Belo Horizonte, the IVS has served as one of many ways of understanding local realities to guide public health policies and prioritize resource allocation. The present study adds new analyses of the frequency of a major risk factor for NCDs and intraurban inequalities [32].

The literature shows that low income and schooling are associated with a higher prevalence of tobacco use both in Brazil [33, 34] and in other countries [35] and with increasing and increased nicotine dependence [36]. This fact was confirmed in the present study, where the prevalence of adult smokers living in high-risk areas was higher than the prevalence among those living in low-risk areas containing populations with better socioeconomic circumstances.

This study has some limitations. First, we had to exclude 14% of the Vigitel interviews due to the inability to identify the census tract through linkage. Second, in the set of census tracts without Vigitel interviews, information about adult smokers or non-smokers was added. Third, the post-stratification weights were used according to the population of the 2010 census by the IVS to minimize potential selection biases due to the absence of census tracts without interviews and for joint analyses of interviews by period. Fourth, the differences found between the estimates obtained by the direct and indirect methods require internal and external validation of the results found in this article and thus validate the methodology adopted to estimate prevalence at the small area level using the Vigitel data.

## Conclusions

The most socioeconomically disadvantaged population is more affected by risk factors for chronic diseases, such as smoking, becoming more ill and having poorer access to health services, which further increases the inequality that affects Brazil. This study found differences in the profile of smokers by the IVS, suggesting that information about subgroups such as these will assist in the formulation, implementation, monitoring and evaluation of the impact of public health policies targeting smoking, especially when estimated by means of adequate small area methodology. These findings can contribute to guiding public policies to define priorities for resource allocation and identifying more vulnerable populations.

## Data Availability

Part of the data is public and available for consultation on the site: http://svs.aids.gov.br/download/Vigitel/. The data sets generated and/or analysed during the current study are not available to the public because they contain census tracts and cannot be accessed because they are sensitive data that may lead to the identification of housing. However, they are available from the corresponding author upon reasonable request.

## Abbreviations

CNEFE: National Register of Addresses for Statistical Purposes
HDI: Human Development Index
IBGE: Brazilian Institute of Geography and Statistics
IVS: Health Vulnerability Index
NCD: Non-communicable diseases
SDI: Sociodemographic Index
Vigitel: Surveillance System for Risk Factors and Protection for NCD
WHO: World Health Organization
